# Agriculture Alters Gonadal Form and Function in the Toad *Bufo marinus*

**DOI:** 10.1289/ehp.11536

**Published:** 2008-07-03

**Authors:** Krista A. McCoy, Lauriel J. Bortnick, Chelsey M. Campbell, Heather J. Hamlin, Louis J. Guillette, Colette M. St. Mary

**Affiliations:** 1 School of Natural Resources and Environment, University of Florida, Gainesville, Florida, USA; 2 Department of Zoology, University of Florida, Gainesville, Florida, USA

**Keywords:** amphibians, endocrine disruption, intersex, pesticides, secondary sexual traits

## Abstract

**Background:**

Many agricultural contaminants disrupt endocrine systems of wildlife. However, evidence of endocrine disruption in wild amphibians living in agricultural areas has been controversial. Typically, studies on the effects of pollutants on wildlife attempt to compare polluted with unpolluted sites.

**Objectives:**

We took a novel approach to address this question by explicitly quantifying the relationship between gonadal abnormalities and habitats characterized by differing degrees of agricultural activity.

**Methods:**

We quantified the occurrence of gonadal abnormalities and measures of gonadal function in at least 20 giant toads (*Bufo marinus*) from each of five sites that occur along a gradient of increasing agricultural land use from 0 to 97%.

**Results:**

The number of abnormalities and frequency of intersex gonads increased with agriculture in a dose-dependent fashion. These gonadal abnormalities were associated with altered gonadal function. Testosterone, but not 17β-estradiol, concentrations were altered and secondary sexual traits were either feminized (increased skin mottling) or demasculinized (reduced forearm width and nuptial pad number) in intersex toads. Based on the end points we examined, female morphology and physiology did not differ across sites. However, males from agricultural areas had hormone concentrations and secondary sexual traits that were intermediate between intersex toads and non-agricultural male toads. Skin coloration at the most agricultural site was not sexually dimorphic; males had female coloration.

**Conclusions:**

Steroid hormone concentrations and secondary sexual traits correlate with reproductive activity and success, so affected toads likely have reduced reproductive success. These reproductive abnormalities could certainly contribute to amphibian population declines occurring in areas exposed to agricultural contaminants.

Exposure to endocrine-disrupting chemicals (EDCs), which alter normal hormonal signaling during embryonic development, can permanently change adult reproductive system morphology and function, as well as reproductive behavior (Guillette et al. 1995; McLachlan 2001; Zala and Penn 2004). Moreover, many components of the endocrine system are highly conserved, so EDCs can affect normal physiological functions across diverse taxa (Crews and McLachlan 2006; McLachlan 2001). Indeed, many studies have shown that exposure to EDCs is responsible for changes in anatomy, behavior, and reproductive system function in a wide array of vertebrate taxa, including fishes (Jobling et al. 2006), amphibians (Hayes et al. 2003), reptiles (Guillette and Iguchi 2003), and mammals (Sonne et al. 2006), including humans (Guillette et al. 2006; Swan 2006).

Despite this growing body of literature documenting endocrine disruption, much controversy remains about whether reproductive abnormalities observed in wild populations are caused by EDCs and not some other natural process. This controversy is fueled, in large part, by the difficulty of directly linking endocrine-disrupting effects identified in laboratories with field observations. Wild organisms are exposed to complex mixtures of chemicals (Hayes et al. 2006) and other environmental conditions (Edwards et al. 2006) that can obfuscate a causal link between abnormalities and a particular toxic agent or generate novel pathologies not identified in controlled laboratory studies. Investigating patterns of pathologies in nature is further challenged because many studies are designed to compare polluted sites against reference sites, yet there are likely no sites on Earth that are not affected by pollution. For example, agricultural pesticides that are known endocrine disruptors are transported hundreds of miles through air before being deposited by precipitation into once pristine areas such as Isle Royale National Park (Lake Superior) (Nations and Hallberg 1992; Thurman and Cromwell 2000).

Many recent laboratory and field studies have suggested that agricultural contaminants are associated with amphibian reproductive abnormalities and population declines (Davidson and Knapp 2007; Ouellet et al. 1997; Sparling et al. 2001). Hayes et al. (2003) found higher incidences of gonadal abnormalities and hermaphroditism (intersex: male and female gonadal tissue in the same individual) in frogs exposed to the herbicide atrazine in the laboratory and from those collected from atrazine-contaminated agricultural areas relative to reference sites, and they suggested that EDC-induced reproductive abnormalities could contribute to the global decline of amphibian populations. Indeed, Davidson and Knapp (2007) recently showed that windborne pesticides are one causal factor driving amphibian population declines in some locations by demonstrating that the degree of protection from windborne pesticides was a significant predictor of *Rana muscosa* distributions.

Other researchers have not found such strong relationships between agriculture and reproductive abnormalities, and they argue that observed gonadal malformations result from natural developmental processes. For instance, Murphy et al. (2006) found no consistent relationship between agricultural contaminants (including atrazine) and the incidence of hermaphroditism or testicular oocytes. In their study, frogs with testicular oocytes were found at both agricultural (polluted) and nonagricultural (reference) sites (Murphy et al. 2006). None of the amphibian species reported by Murphy et al. (2006) are known to be naturally hermaphroditic or to commonly have testicular oocytes as adults (Hayes 1998). However, Murphy et al. (2006) reported that 25–50% of adults from three different species had testicular oocytes. Such proportions of testicular oocytes in naturally gonochoristic adults are not likely to be the result of a rare genetic anomaly. It is likely that the sites categorized as nonagricultural by Murphy et al. (2006) were nevertheless polluted with some type(s) of EDC.

In the present study, we took a novel approach to asking whether gross gonadal abnormalities of an anuran amphibian species (*Bufo marinus*) are associated with agricultural exposure. Unlike most previous studies, we explored this question across a range of sites that vary in degree of agricultural intensity. In fact, the proportion of agriculture at our study sites was inversely related to the degree of urbanization and its associated stressors. Therefore, we explicitly tested whether gonadal abnormalities are randomly distributed across human-modified landscapes or are associated with increasing exposure to agriculture specifically.

We surveyed *B. marinus* across five sites that differed in degree of agricultural activity and determined the total number and types of gonadal abnormalities as well as the incidence of intersex individuals across this continuum. We found a strong increase in gonadal abnormalities as agricultural activity increased, so we went further to determine if the gonads of toads living in agricultural areas had altered function relative to the gonads of nonagricultural toads. We compared circulating sex steroid concentrations, and as more integrated signals of seasonal endocrine system function, we compare three sex-hormone–dependent secondary sexual characteristics.

## Materials and Methods

### Study sites and collection techniques

We surveyed five sites in south Florida that varied in habitat type from completely suburban to completely agricultural. To quantify the intensity of agricultural exposure, we imported Google Earth (Google Inc., Mountain View, CA) digital satellite images and a scale into the Image Pro Plus image analysis program (Image House A/S, Copenhagen, Denmark) on 20 August 2007 and calculated the total percentage of agricultural land (vegetable and sugarcane farming, no livestock) within a 5.6-km^2^ area around each collection site. The home range of *B. marinus* is approximately 2 km^2^ (Zug et al. 1975), so this measure reflects the area experienced by collected toads.

We then assigned sites an “agricultural intensity” rank of 1–5 based on the percentage of agriculture in the 5.6-km^2^ area around the site or its proximity to agriculture when no agricultural land (0%) fell within the 5.6-km^2^ area. We used these ranks for all Kendall’s tau tests of association. Lake Worth (LW) and Wellington (WT) contained no agriculture (0%). LW was 22 km away from an agricultural area and was ranked 1, whereas WT was 5.18 km away from agriculture, and was ranked 2. Homestead (HS) contained 34% agricultural land, which we ranked 3. Canal Point (CP; 51%, bordered on one side by Lake Okeechobee) and Belle Glade (BG; 97%) contained the most agricultural land; these were ranked 4 and 5, respectively.

Although the presence of specific contaminants at each type of site certainly overlapped, urbanized and agricultural habitats are polluted with different milieus of chemicals that are expected to induce different suites of pathologies. Polyaromatic hydrocarbon concentrations are ubiquitous in urbanized areas (Standley et al. 2000), whereas agricultural areas are more typically polluted with pesticides (Crosbie and Chow-Fraser 1999).

Twenty or more adult toads (> 91 mm body length) (Cohen and Alford 1993; Meshaka et al. 2006) were collected from each site over a 2-year period. Year had no effect on the percentage or types of abnormalities documented at sites where collections occurred in more than 1 year.

Toads from all locations were collected at night, while feeding, under mercury vapor streetlights. Each individual was euthanized with an overdose of the anesthetic 0.3% tricaine methanesulfonate at pH 7 (Sigma-Aldrich, St. Louis, MO), photographed, and measured for snout–vent length (nearest 1 mm). Approximately 3 mL of whole blood was collected via cardiac puncture and centrifuged. Plasma samples were frozen at −20°C, transported back to the laboratory on dry ice, and stored at −80°C until hormone assays were conducted. One gonad from each individual was fixed in neutral buffered formalin for gross morphologic analysis. All procedures were approved by the University of Florida’s institutional animal care and use committee (permit D699); animals were treated humanely, and they did not suffer any pain.

### Analyses of primary sexual characteristics

We evaluated all primary sexual characteristics in two ways. First, we evaluated individuals with each abnormality type across sites with Kruskal-Wallis one-way analysis of variance (ANOVA) to determine if the occurrence of specific abnormalities differed across sites. The Kruskal-Wallis test does not explicitly test whether effects increase with increasing agriculture, so we used a one-tailed Kendall’s tau-b test of association to explicitly test whether site differences in sex group statistically corresponded with agricultural intensity (Siegel and Castellan 1988). We ran all Kruskal-Wallis tests in SPSS 15.0 (SPSS Inc., Chicago, IL), and Kendall’s tau tests in R free statistics software (Wessa 2007).

We observed no gross morphologic abnormalities in females, so all analyses on gonadal morphology include only males, males with Bidder’s organ abnormalities (Bidder’s males), and intersexes. These toads were assigned a sex group category (males = 1, Bidder’s males = 2, intersexes = 3), and differences in the mean sex group across sites were evaluated as described above.

**Figure 1:**
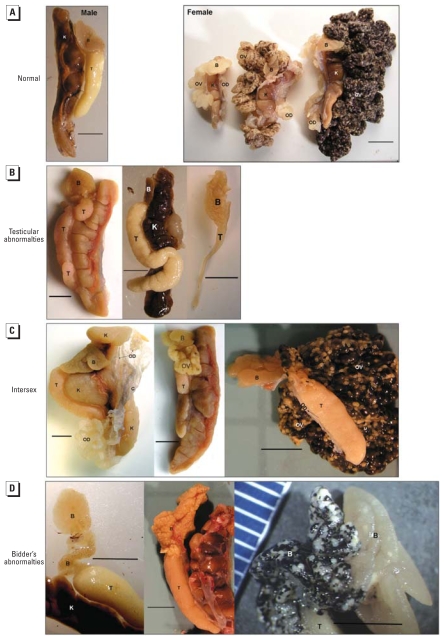
Four broad categories of gonadal phenotypes observed. Abbreviations: B, Bidder’s organ; C, connective tissue; K, kidney; OV, ovary; OD, oviduct; T, testis. (*A*) Toads that had no obvious gross gonadal abnormalities were categorized as having normal gonadal morphology. (Left) “Males” had testes, a nonvitellogenic Bidder’s organ, and no ovaries or oviducts. (Right) “Females” had ovaries, oviducts, nonvitellogenic Bidder’s organs, and no testes; ovaries in various stages of oogenesis were collected (oogenesis and accumulation of vitellogenin increases from left to right). (*B*) Toads that were categorized as “male” in some cases had testicular abnormalities. (Left) Multiple testes. (Center) Abnormally shaped testes. (Right) Abnormally small testes. (*C*) Intersex toads had both testes and oviducts or ovaries. (Left) Well-developed oviduct and testis, but no ovary. (Center) Nonvitellogenic ovary that is distinct from the Bidder’s organ and testis. (Right) Intersex with a highly vitellogenic ovary. (*D*) “Bidder’s males” had multiple (left), early vitellogenic (center), or late vitellogenic (right) Bidder’s organs but no ovaries or oviducts. Bars = 0.5 cm.

We also categorized individuals based on quantity and type of gonadal abnormality. We assigned scores and evaluated them based on the total number of abnormalities in each of the following three tissue types: *a*) testes, *b*) ovaries/oviducts in the presence of testes (number and developmental stage of female gonadal tissues found in the presence of testes is considered a measure of the severity of the intersexed condition), and *c*) Bidder’s organs (Figure 1).

In addition, the total number of abnormalities were summed and similarly evaluated to determine if the mean and maximum number of gonadal abnormalities differed among sites and corresponded to increased agricultural intensity. As an example of our ranking system, an individual with misshaped testes (one abnormality in the testes category), a vitellogenic Bidder’s organ (one abnormality in the Bidder’s organ category), and ovaries (in the presence of testes) that were also vitellogenic (two abnormalities in the ovary/oviduct category) received a score of 4.

### Analyses of gonadal function

Our next objective was to determine if toads from the most agricultural areas had altered gonadal function relative to those from nonagricultural areas and to assess whether intersex and Bidder’s males from agricultural sites were feminized and demasculinized relative to males from the same site and those from nonagricultural areas. In fact, not all gonadal groups occurred at every site (no intersex toads occurred at nonagricultural sites), so we could not compare gonadal groups across all sites. Therefore, for each measure of gonadal function, we grouped toads into six different sex categories based on gross gonadal morphology and their occurrence in nonagricultural (LW, WT) or agricultural (BG, CP) sites: *a*) non-agricultural females (NAgFs), *b*) agricultural females (AgFs), *c*) agricultural intersex toads (intersexes), *d*) agricultural Bidder’s males (Bidder’s males), *e*) agricultural males (AgMs), and *f* ) nonagricultural males (NAgMs). We did not include toads from the intermediate agricultural intensity site (HS, 34%) in these analyses because they could not be considered as NAgM or as AgMs.

### Sex hormone concentrations

We analyzed circulating 17β-estradiol (E_2_) and testosterone concentrations with a validated radio-immunoassay using the 96-well FlashPlate PLUS system (PerkinElmer, Waltham, MA). Data were log_10_ transformed to meet the assumptions of normality and homogeneity of variances and then analyzed using a linear mixed model, where assay (plate) and assay by sex interaction were identified as the random effects. We used SPSS default post hoc comparisons (least significant difference). E_2_ to testosterone ratios (E_2_/testosterone) were log_10_ transformed to meet the assumptions of normality and homogeneity of variances and evaluated using a one-way ANOVA. We conducted post hoc comparisons using Tukey’s test.

### Secondary sexual characteristics

We used photographs to compare body color pattern, number of nuptial pads, and forelimb size in relation to the six sex groups identified above. We renamed all photographs, and observers were blind to the sex and site from which the toads were collected. We analyzed all statistics using SPSS 15 and conducted post hoc comparisons using Tukey’s tests.

### Color pattern

We quantified the number of color changes (mottling) that occurred across a transect line drawn from the left eye to the vent of each photographed toad using Image Pro Plus and compared mottling across sex groups using a one-way ANOVA. In addition, we analyzed male and female mottling scores across sites separately using a two-way ANOVA to test for changes in color dimorphism across sites (sex × site).

### Forelimb size

We measured the width of the forelimb across the radioulna distal to the humerus and perpendicular to the arm axis using Image Pro Plus and tested for differences among sex groups using an analysis of covariance (ANCOVA), with sex group as the fixed effect, log_e_(forelimb width) as the dependent factor, and log_e_(body length) as the covariate to account for the influence of body size. We conducted natural log transformations to linearize the data. All slopes were homogeneous; thus, we removed the interaction from the analysis and used the simplified ANCOVA to estimate marginal means for the elbow width of each group. We compared sex groups at a snout-vent length of 120.2 mm.

### Nuptial pads

No females had nuptial pads and thus were not included in this analysis. We used one-way ANOVA to determine if the mean number of nuptial pads per limb was different among sex groups.

## Results

### Primary sexual characteristics

**Figure 2:**
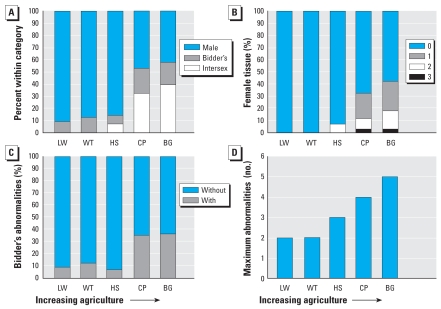
Gonadal abnormalities increase with increasing agriculture in a dose-dependent manner. (*A*) Percentage of individuals classified as males, males with Bidder’s organ abnormalities (Bidder’s), or intersexes at each site. (*B*) Percentage of individuals with testes and a specific number of female tissues or advanced stage of oogenesis; the number of female tissues found in toads with testes (0–3) is a measure of the severity of the intersex condition. (*C*) Percentage of toads with (1) or without (0) Bidder’s organ abnormalities at each collection site. (*D*) Maximum number of gonadal abnormalities at each site. Agricultural intensity increases along the x-axis from left to right for each graph.

Mean sex group rank increased as a function of agriculture. More intersex individuals (Figure 1C) occurred at the two most agricultural sites than at the other sites (Kruskal-Wallis_4_ = 21. 6, *p* < 0.001; Kendall’s tau-b_3_ = 1, *p* = 0.01; Figure 2A). Testicular abnormalities (Figure 1B), such as misshaped testes, were relatively common and did not vary across sites or increase with agricultural intensity (KW_4_ = 5.1, *p* = 0.272; Kendall’s tau-b_3_ = 0.4, *p* = 0.23). However, the number of female-tissue abnormalities (e.g., ovaries or oviducts in the presence of testes) were significantly different among sites and increased with increasing agricultural intensity (KW_4_ = 17.3, *p* = 0.002; Kendall’s tau-b_3_ = 0.9, *p* = 0.02; Figures 1C, 2B). Importantly, the number and developmental stage of female gonadal tissues found in the presence of testes are considered a measure of the severity of the intersexed condition. Applying that measure, toads became more severely intersexed as agriculture increased. Bidder’s organ abnormalities tended to vary across sites and was highest at the agricultural sites (KW_4_ = 9.0, *p* = 0.06; Figures 1D, 2C) but did not correspond significantly to increasing agriculture (Kendall’s tau-b_3_ = 0.6, *p* = 0.1; Figures 1D, 2C). The total number of gonadal abnormalities increased with increasing agriculture (KW_4_ = 21.2, *p* < 0.001; mean Kendall’s tau-b_3_ = 0.8, *p* = 0.04; maximum Kendall’s tau-b_3_ = 0.9, *p* = 0.02; Figure 2D).

### Sex hormone concentrations

**Figure 3:**
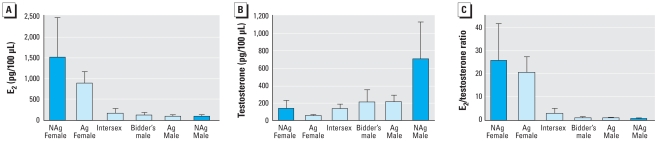
Sex hormone concentrations of toads from nonagricultural and agricultural sites separated by sex group. (*A*) E_2_ concentrations. (*B*) Testosterone concentrations. (*C*) E_2_/testosterone ratio across sex groups. NAg, toads collected from nonagricultural areas; all other toads were collected from agricultural areas. Error bars are 95% confidence intervals around the mean of the untransformed data. Statistical analyses were conducted on transformed values.

We found significant differences in plasma E_2_ concentrations among sex groups (*F*_5,157_ = 23.74, *p* < 0.001), but these differences were driven entirely by high E_2_ concentrations in females relative to all other sex groups (Figure 3A). AgFs and NAgFs were not significantly different from one another.

Plasma testosterone concentrations were also significantly different among sex groups (*F*_5,162_ = 11.26, *p* < 0.001; Figure 3B). NAgFs and AgFs did not differ in circulating testosterone concentrations, but had significantly lower testosterone levels than did NAgMs. Intersex toads had plasma testosterone concentrations that were similar to NAgFs and were not different from Bidder’s males and AgMs, but were significantly lower than NAgMs. Both Bidder’s males and AgMs were significantly different from females, but only AgMs had significantly lower testosterone levels relative to NAgMs (Figure 3B).

The ratio of E_2_ to testosterone concentration (E_2_/testosterone) was significantly different among sex groups (*F*_5,150_ = 48.46, *p* < 0.001; Figure 3C). Females were not significantly different from one another but had higher E_2_/testosterone ratios relative to all other groups. The E_2_/testosterone ratio of intersex toads was significantly lower than that in both groups of females and similar to Bidder’s males and AgMs, but was higher than NAgMs. On average, intersex toads had more E_2_ than testosterone (mean E_2_/testosterone ratio > 1; Figure 3C). Bidder’s males and AgMs were intermediate between and not significantly different from intersexes and NAgMs (Figure 3C).

### Color pattern

**Figure 4:**
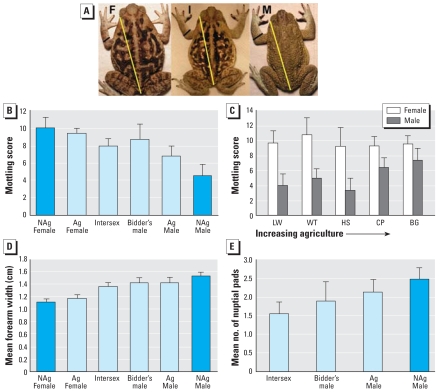
Sexually dimorphic secondary sexual traits are altered at agricultural sites. (*A*) Photographs of female (F), intersex (I), and male (M) toads. We measured mottling by counting the number of color changes that occurred along the yellow transect line, and the portion of the forelimb that was measured and compared is shown as a black line. Males can have nuptial pads on the first three digits, whereas females have none. (*B*) Mean mottling score across each individual’s body separated by sex group. (*C*) Mean mottling score for females and males across sites; agricultural intensity increases along the x-axis from left to right. (*D*) Mean body-size–corrected forelimb width for each sex group. (*E*) Mean number of nuptial pads by sex groups. NAg, toads collected from nonagricultural areas; all other toads were collected from agricultural sites. Error bars indicate 95% confidence intervals.

Mottling score varied as a function of sex category (*F*_5,128_ = 15.6, *p* < 0.001; Figure 4B). NAgFs and AgFs were highly mottled and similar. Intersexes and Bidder’s males were similar in mottling to females and significantly more mottled than NAgMs. AgMs were less mottled than females but more mottled than NAgMs. NAgMs were the only group that was significantly less mottled than all other sex groups.

We found a significant interaction between sex and site on mottling (*F*_4,103_ = 2.7, *p* = 0.03; Figure 4C). Although female mottling did not change across sites, the sexual dimorphism in mottling became less pronounced as agricultural intensity increased because males were feminized in coloration (Figure 4C).

### Forelimb size

Mean forelimb width was significantly different among sex groups (*F*_5,70_ = 33.8, *p* < 0.001; Figure 4D). Females had smaller forelimbs than any other group. Intersex individuals had arm widths that were intermediate between and significantly different from females and NAgMs. Bidder’s males and AgMs had larger forelimbs than did females but were intermediate between and not significantly different from intersexes or NAgMs.

### Nuptial pads

Nuptial pad number was significantly different among sex groups (we did not consider females in this analysis) (*F*_3,76_ = 6.0, *p* = 0.001; Figure 4E). Intersex toads had significantly fewer nuptial pads than did NAgMs, whereas numbers of nuptial pads among Bidder’s males and AgMs were intermediate between and not significantly different from those of either intersex or NAgMs.

## Discussion

Here we show, for the first time, that reproductive abnormalities in primary sexual traits of wild (feral) *B. marinus* are statistically associated with agricultural activity and increase with percentage of agricultural exposure in a dose-dependent manner. The severity of intersexuality and the maximum number of gonadal abnormalities increase with agricultural intensity. Gonadal abnormalities, such as those reported here, are likely to reduce the reproductive success of affected individuals and could help explain why independent studies have documented that amphibian populations exposed to pesticides are declining or have gone extinct (e.g., Davidson and Knapp 2007; Sparling et al. 2001).

Furthermore, we have documented the alteration of sexually dimorphic external morphologic traits that makes *B. marinus* an ideal sentinel species for studying the effects of EDCs. These traits serve as visual “tags” advertising that an individual has experienced endocrine disruption. *B. marinus* are widely distributed and therefore can be studied at very large scale to provide important information about the distribution of abnormalities associated with various forms of land use including a diversity of agricultural uses.

Toads from agricultural sites in this study have gonadal deformities that clearly alter gonadal function. Accumulation of vitellogenin(s) within the Bidder’s organ in males is only known to occur after castration, which has led to the conclusion that the testes are necessary in males to suppress accumulation of vitellogenin(s) within the Bidder’s organ (Brown et al. 2002; Pancak-Roessler and Norris 1991; Zaccanti et al. 1994). We found that approximately 20% of the individuals at the agricultural sites have vitellogenic oocytes within their Bidder’s organs, suggesting that the testes of these toads are not functioning normally to suppress Bidder’s organ oogenesis. We also found that naturally sexually dimorphic secondary sexual traits such as skin color, forearm width, and nuptial pad number are altered in toads from agricultural areas, again suggesting that testicular function is compromised in toads from these sites.

Sexual characteristics are maintained via endocrine signaling and are typically modulated via sex steroids produced in the gonads (Hayes and Menendez 1999). Female coloration in the reed frog (*Hyperolius argus*) is estrogen dependent, and early induction of female color pattern has been proposed as a biomarker for estrogenic activity (Noriega and Hayes 2000). The dark and mottled skin coloration typical of female *B. marinus* is due to dense melanin concentrations that are modulated in mammals, at least in part, by estrogens (McLeod et al. 1994; Shah and Maibach 2001; Slominski et al. 2004; Thorton 2002). Therefore, we hypothesized that color pattern differences function as a biomarker of endogenous estrogen concentration. However, E_2_ concentrations were not significantly different between highly mottled intersex toads and less mottled NAgMs. Although the loss of sexually dimorphic coloration patterns in *B. marinus* is associated with increased agriculture, there does not seem to be a clear association between plasma E_2_ concentrations and increased mottling. Plasma testosterone concentrations were reduced in all agricultural toads (with testes) as was male-typical coloration, so sexually dimorphic coloration could be associated with testosterone rather than E_2_ concentrations in *B. marinus*. Alternatively, expression of P450 aromatase in the skin and local aromatization of androgens to estrogens could be responsible for body color variation.

Furthermore, forearm width and nuptial pad number are androgen dependent and function in mating (Emerson et al. 1999; Epstein and Blackburn 1997; Lynch and Blackburn 1995; Rastogi and Chieffi 1971; Thomas and Licht 1993; Thomas et al. 1993; van Wyk et al. 2003; Wetzel and Kelley 1983). We show that these traits are reduced in intersex individuals, suggesting that intersex toads do not build hypertrophied musculature or develop nuptial pads typically associated with normal levels of androgens and properly functioning testes (Lee and Correales 2002). Indeed, intersex toads have reduced testosterone concentrations, more similar to females than to NAgMs. Thus, arm width and nuptial pad number are biomarkers of androgen status. Because nuptial pad development is seasonal, and nonreproductive toads do not possess well-developed nuptial pads, decreased nuptial pad development in intersex animals suggests that they are not as reproductively active as NAgMs. Moreover, nuptial pads and forearm width are under sexual selection (Lee and Correales 2002). Thus, the abnormalities found in this study also likely influence the nature and action of sexual selection.

The occurrence of intersex individuals could arise through feminization of males or masculinization of females. If females were masculinized, we would have found individuals with relatively complete female reproductive tracts and some combination of male traits (e.g., nuptial pads, decreased mottling, wider arms, small testes). We did not find this. Instead, intersex individuals typically had normal-sized testes, but varied in ovary size and stage of oogenesis. In addition, no females (ovaries and oviducts, but no testes) had nuptial pads or solid coloration. Females did not differ in color pattern across sites, but males were more mottled (feminized coloration) in agricultural areas, and we did not observe the typical sexual dimorphism in mottling at the most agriculture site—most toads were mottled (Figure 4C). Therefore, male *B. marinus* are feminized and demasculinized at agricultural sites.

Many of the gonadal abnormalities associated with agriculture in this study are organizational and remain throughout the toads’ adult lives. Intersex gonads consisted of distinct ovarian and testicular tissues that were not always bilaterally similar, suggesting that reproductive development is fundamentally altered. This is similar to other studies that have reported both ovarian and testicular tissue in frogs obtained from agricultural areas or after exposure of tadpoles to atrazine (Hayes et al. 2003). These observations are not exclusive to agricultural areas in other studies (Murphy et al. 2006), but differences in study design (e.g., how sites are defined) and potential differences in the responsiveness of subpopulations could account for some of these differences. An important question remains concerning the frequencies at which such abnormalities occur under “normal” environmental conditions. Our study does not explicitly address this because we did not examine populations with little human impact. However, we found that the frequency of the intersex condition increased with agricultural activity and was absent in areas with zero agriculture, suggesting that intersexes do not occur at high frequencies in areas unaffected by agriculture.

The timing, amount, mixture, and fluctuation of contaminant concentrations are important factors that determine phenotypic effects (Edwards et al. 2006; Guillette et al. 1995; Milnes et al. 2006). Therefore, a single mechanism of toxicity, identified in controlled laboratory settings, is unlikely to be acting alone under natural conditions. Thus, we should not expect to identify chemical-specific mechanisms of toxicity occurring in wild animals. A few of the pesticides used at our agricultural sites cause endocrine disruption and have well-studied mechanisms of toxicity. Glyphosate (Roundup) and atrazine are used at CP and BG and are known to disrupt steroidogenesis (Oliveira et al. 2007; Soso et al. 2007). Glyphosate disrupts steroidogenic acute regulatory (StAR) protein expression across diverse taxonomic groups, which modulates the initial step in the steroidogenic pathway (Walsh et al. 2000) and leads to reductions in steroid hormone production, including both testosterone and E_2_. Research conducted across several vertebrate classes has shown that atrazine exposure can inhibit androgen production and increase estrogens by increasing aromatase transcription (Crain et al. 1997; Fan et al. 2007; Sanderson et al. 2002). We found that testosterone is modulated among sex groups but E_2_ is not. It is still possible that other estrogens that we have not measured vary across sex groups. As we might expect, hormone concentrations in our feral toads are not entirely consistent with the patterns of toxicity documented in the laboratory for either of these well-studied compounds. Altered phenotypes, including hormone concentrations, in this study are a result of multiple exposures to several chemicals at various concentrations over the lifetime of the toads.

We found that gonadal form and function in the anuran amphibian *B. marinus* are altered by agricultural land use in a dose-dependent fashion. Therefore, our study shifts the focus of the current literature debate from asking if gonadal abnormalities are associated with agriculture to a new line of questions focused on identifying affected species, chemical causes, and developmental, physiological, and ecological implications of exposures. Pesticides are distributed globally and have been explicitly linked to amphibian population declines (Davidson and Knapp 2007; Sparling et al. 2001). It is likely that contaminant-induced reproductive abnormalities contribute to such declines.
